# Circular RNA circ_0003423 Promotes Osteoarthritis Progression by Sponging miR‐330‐5p to Upregulate TWIST1‐Mediated Chondrocyte Inflammation and Extracellular Matrix Degradation

**DOI:** 10.1111/jcmm.71278

**Published:** 2026-07-06

**Authors:** Lei Zhang, Hua Wang, Shaoyang Liu, Jianjun Qiu, Yue Ding, Fei Wu

**Affiliations:** ^1^ Department of Orthopaedics Putuo Hospital, Shanghai University of Traditional Chinese Medicine Shanghai China; ^2^ Department of Orthopedics Putuo People's Hospital, School of Medicine, Tongji University Shanghai China

**Keywords:** chondrocytes, circ_0003423, miR‐330‐5p, osteoarthritis, TWIST1

## Abstract

Osteoarthritis (OA) is a debilitating degenerative joint disease characterized by progressive articular cartilage destruction, persistent inflammation, and functional impairment. Circular RNAs (CireRNAs) have emerged as critical epigenetic regulators in OA pathogenesis; however, the precise functional role of Circ_0003423 in OA remains largely unexplored. This study aimed to elucidate the expression pattern, biological functions, and underlying molecular mechanism of Circ_0003423 in OA progression. Bioinformatic analysis of the GSE178724 dataset was performed to identify differentially expressed CircRNAs in OA cartilage. Circ_0003423 and miR‐330‐5p expression levels were quantified by QRT‐PCR in IL‐1β–stimulated chondrocytes. The interaction between Circ_0003423 and miR‐330‐5p, and between miR‐330‐5p and TWIST1, was validated by RNA pull‐down and dual‐luciferase reporter assays. Western blotting was employed to assess TWIST1, Collagen I, Collagen II, ADAMTS5, MMP3, MMP13, total NF‐κB p65, and phosphorylated NF‐κB p65 (p‐NF‐κB p65) expression. Chondrocyte proliferation and apoptosis were evaluated by CCK‐8 and EdU assays and flow cytometry, respectively. Pro‐inflammatory cytokine levels were measured by ELISA. An OA mouse model was established via destabilization of the medial meniscus (DMM), and Circ_0003423 was silenced by intra‐articular injection of lentiviral shRNA to validate In vivo findings. Circ_0003423 was significantly upregulated in OA cartilage and IL‐1β–stimulated chondrocytes. Silencing Circ_0003423 rescued IL‐1β–induced proliferation inhibition, reduced apoptosis, attenuated pro‐inflammatory cytokine secretion, and mitigated ECM degradation. Mechanistically, Circ_0003423 functioned as a ceRNA by directly sponging miR‐330‐5p, which targeted TWIST1 to activate downstream NF‐κB signalling and upregulate ADAMTS5, MMP3, and MMP13, with concomitant collagen downregulation. Rescue experiments confirmed that miR‐330‐5p inhibition or TWIST1 overexpression abrogated the protective effects of circ_0003423 silencing. In vivo, circ_0003423 silencing upregulated miR‐330‐5p, suppressed TWIST1/NF‐κB/MMP3/MMP13 activation, and alleviated cartilage degeneration, chondrocyte apoptosis, and inflammatory responses in DMM mice. Circ_0003423 drives OA progression by functioning as a ceRNA to sponge miR‐330‐5p, thereby de‐repressing TWIST1 and activating downstream NF‐κB signalling and MMP3/MMP13‐mediated ECM destruction. Targeting circ_0003423 represents a promising therapeutic strategy for OA intervention.

## 1 Introduction

Osteoarthritis (OA) is a prevalent and chronic degenerative joint disorder marked by articular cartilage degradation, progressive structural damage, and sustained inflammatory responses, collectively rendering it one of the leading causes of disability and diminished quality of life among the elderly population [1, 2]. In the context of global population aging, the incidence of OA is rising annually, placing an escalating burden on healthcare systems worldwide [3, 4]. Despite decades of research, current therapeutic strategies, including pharmacological treatments, physical rehabilitation, and surgical interventions, merely provide symptomatic relief but fail to halt disease progression or reverse cartilage destruction at the biological level [5, 6]. Consequently, a deeper understanding of the molecular mechanisms underlying OA pathogenesis is urgently needed to identify novel and effective therapeutic targets that can ultimately improve disease management and long‐term patient outcomes.

In recent years, non‐coding RNAs (ncRNAs) have emerged as pivotal regulators in diverse human diseases [7]. Among them, circular RNAs (circRNAs) represent a distinct and unique class of ncRNAs characterized by a covalently closed loop structure, which confers remarkable stability and evolutionary conservation [8, 9]. CircRNAs are well‐established to regulate gene expression by acting as microRNA (miRNA) sponges, thereby modulating various biological processes including proliferation, apoptosis, and inflammatory signalling [10, 11]. A growing body of evidence indicates that specific circRNAs are aberrantly expressed in OA. Of particular interest, circ_0003423, which was previously implicated in atherosclerosis, has recently been suggested to play a pivotal role in OA pathogenesis [12, 13]. Concurrently, dysregulated miRNAs in OA are closely associated with chondrocyte apoptosis, inflammation, and extracellular matrix (ECM) degradation. Notably, miR‐330‐5p, which is consistently downregulated in OA, has been suggested to influence disease progression through the regulation of critical downstream target genes [14]. TWIST1, a transcription factor involved in numerous biological processes, is aberrantly upregulated in OA, contributing to inflammatory responses and ECM degradation in chondrocytes [15, 16, 17]. Yet, whether circRNAs regulate TWIST1 expression through miRNA‐mediated mechanisms in OA remains largely unclear.

While circRNAs and miRNAs have been implicated in OA, the precise functional role and mechanistic contribution of circ_0003423 to OA pathogenesis have not been fully elucidated. Key questions remain unanswered as to whether circ_0003423 promotes OA progression by sponging miR‐330‐5p, and whether this ceRNA regulatory network engages TWIST1 as a critical downstream effector to drive chondrocyte dysfunction, NF‐κB‐mediated inflammation, and MMP‐dependent ECM destruction. Moreover, the majority of studies have been confined to In vitro settings, with insufficient validation in animal models, thereby restricting a comprehensive understanding of circ_0003423 function in OA.

The present study was therefore designed to investigate the functional role and molecular mechanism of circ_0003423 in OA pathogenesis. By integrating bioinformatic analysis of publicly available sequencing data from OA cartilage samples with experimental validation in IL‐1β‐stimulated chondrocytes, we characterized the expression pattern of circ_0003423 and explored whether it acts as a ceRNA to modulate the miR‐330‐5p/TWIST1 axis, thereby regulating chondrocyte proliferation, apoptosis, NF‐κB‐driven inflammation, and MMP‐mediated ECM degradation. The therapeutic relevance of circ_0003423 knockdown was further validated in a DMM‐induced OA mouse model. Collectively, this study elucidates the circ_0003423/miR‐330‐5p/TWIST1/NF‐κB regulatory network, providing mechanistic insights into OA pathogenesis and offering a theoretical foundation for the development of circRNA‐based therapeutic strategies against OA.

## 2 Methods

### 2.1 Cell Culture and Treatment

The human chondrocyte cell line was obtained from the Cell Bank of the Chinese Academy of Sciences Type Culture Collection. Cells were maintained in Dulbecco's Modified Eagle Medium (DMEM; Gibco, USA) supplemented with 10% fetal bovine serum (FBS; Gibco, USA) at 37°C in a humidified atmosphere containing 5% CO_2_ . The culture medium was refreshed every 2–3 days, and cells were subcultured at a 1:3 ratio upon reaching 80%–90% confluence. To establish an In vitro OA model, cells were treated with 10 ng/mL recombinant human IL‐1β (PeproTech, USA) for 24 h. For gene silencing experiments, transfections of negative control siRNA (si‐NC; GenePharma, China) or siRNA targeting circ_0003423 (si‐circ_0003423; GenePharma, China) were performed using Lipofectamine 3000 (Invitrogen, USA) according to the manufacturer's protocol. Experimental groups were designated as follows: (i) Control: cells cultured under standard conditions; (ii) IL‐1β: cells stimulated with IL‐1β alone; (iii) IL‐1β + si‐NC: IL‐1β‐stimulated cells transfected with si‐NC; and (iv) IL‐1β + si‐circ_0003423: IL‐1β‐stimulated cells transfected with si‐circ_0003423. For TWIST1 overexpression experiments, a pLV‐TWIST1 lentiviral expression vector (GenePharma, China) was transfected into chondrocytes, with the empty pLV vector serving as the negative control. All functional assays were performed at 48 h post‐transfection.

### 2.2 qRT‐PCR for circ_0003423 and miR‐330‐5p Expression

#### 2.2.1 RNA Extraction and Reverse Transcription

Total RNA was extracted from cultured chondrocytes using TRIzol reagent (Invitrogen, USA) in accordance with the manufacturer's protocol. RNA concentration and purity were assessed spectrophotometrically using a NanoDrop 2000 spectrophotometer (Thermo Fisher Scientific, USA). First‐strand cDNA synthesis was subsequently performed using the PrimeScript RT kit (Takara, Japan) with the following thermal conditions: 37°C for 15 min, 85°C for 5 s, followed by a final hold at 4°C.

#### 2.2.2 qRT‐PCR

Gene expression levels were quantified by qRT‐PCR using SYBR Green Master Mix (Applied Biosystems, USA) on a StepOnePlus Real‐Time PCR System (Applied Biosystems, USA). Each 20 μL reaction mixture comprised 10 μL SYBR Green Master Mix, 0.4 μL of each forward and reverse primer (10 μM), 2 μL cDNA, and 7.2 μL nuclease‐free water. Thermal cycling conditions consisted of an initial denaturation at 95°C for 30 s, followed by 40 cycles of denaturation at 95°C for 5 s and a combined annealing/extension step at 60°C for 30 s. Relative expression levels were calculated using the 2^−ΔΔCt method, with all reactions performed in triplicate. GAPDH served as the internal reference for circ_0003423 quantification, and U6 snRNA was used for miR‐330‐5p normalization.

### 2.3 RNA Pull‐Down Assay

To assess the direct physical interaction between circ_0003423 and miR‐330‐5p, a biotin‐labelled DNA probe designed to be complementary to the back‐splice junction of circ_0003423 (GenePharma, China) was employed. The probe sequences were as follows: circ_0003423 junction probe: 5′‐Biotin‐AGTGCAAGTCTTCTTCCCGTGCTC‐3′; Scrambled negative control probe: 5′‐Biotin‐TCAGGATCCTCTTCGGACTCTAG‐3′. Briefly, cell lysates were prepared in RIPA buffer (Sigma‐Aldrich, USA) and incubated with the biotinylated probe at room temperature for 30 min, followed by the addition of streptavidin‐coated magnetic beads (Invitrogen, USA), which were rotated for an additional 1 h to facilitate capture of probe–RNA complexes. The beads were subsequently washed three times with PBS (pH 7.4) to remove non‐specific binding. Bound RNA was then eluted, and the enrichment of miR‐330‐5p was quantified by qRT‐PCR to validate the specific interaction between circ_0003423 and miR‐330‐5p. A scrambled biotin‐labelled probe served as a negative control to ensure binding specificity.

### 2.4 RNA Immunoprecipitation (RIP) Assay

To further validate the interaction between circ_0003423 and miR‐330‐5p within the RNA‐induced silencing complex (RISC), an anti‐Ago2 RIP assay was performed using a Magna RIP RNA‐Binding Protein Immunoprecipitation Kit (Millipore, USA) according to the manufacturer's instructions. Briefly, chondrocytes were harvested and lysed in complete RIP lysis buffer supplemented with protease and RNase inhibitors. Cell lysates were then incubated overnight at 4°C with magnetic beads pre‐conjugated with either anti‐Ago2 antibody (Abcam, USA) or normal rabbit IgG (Millipore, USA) as a negative control. Following incubation, the beads were washed six times with RIP wash buffer to eliminate non‐specific binding. Bound RNA was subsequently extracted using TRIzol reagent (Invitrogen, USA), and the enrichment of circ_0003423 and miR‐330‐5p in the Ago2 immunoprecipitates was quantified by qRT‐PCR. Significant enrichment of both circ_0003423 and miR‐330‐5p in the anti‐Ago2 group relative to the IgG control group would indicate their co‐localization within the RISC complex, thereby confirming their functional interaction in chondrocytes.

### 2.5 Validation of the Circular Structure of circ_0003423

The circular nature of circ_0003423 was validated through three complementary approaches. First, to verify the back‐splice junction, both divergent and convergent primers were designed spanning the junction site of circ_0003423. PCR amplification was performed using cDNA and genomic DNA (gDNA) as templates, respectively. The divergent primers were expected to yield a specific amplification product from cDNA but not from gDNA, confirming the existence of the back‐splice junction, while convergent primers served as positive controls for both templates. PCR products were resolved and visualized on a 2% agarose gel. Second, to confirm RNase R resistance, total RNA extracted from chondrocytes was divided into two aliquots: one incubated with 3 U/μg RNase R (Epicentre, USA) at 37°C for 30 min and one left untreated as a negative control. Following treatment, PCR amplification with both divergent and convergent primers was performed and products were visualized by agarose gel electrophoresis. PCR amplification using both divergent and convergent primers yielded visible bands in RNase R‐treated cDNA, confirming that circ_0003423 resisted linear RNA degradation. Third, the identity of the back‐splice junction was unambiguously confirmed by Sanger sequencing of the divergent primer‐amplified PCR product, with the resulting sequence aligned to the reference genome to precisely map the junction site.

### 2.6 Luciferase Reporter Assay

To experimentally validate the direct binding between miR‐330‐5p and the 3‐UTR of TWIST1 mRNA, a wild‐type (WT) fragment of the TWIST1 3‐UTR harbouring the predicted miR‐330‐5p binding site was cloned into the pmirGLO dual‐luciferase vector (Promega, USA). A corresponding mutant construct (Mut) in which the putative miR‐330‐5p binding sequences were site‐specifically disrupted by mutagenesis was generated as a negative control. For reporter assays, chondrocytes were co‐transfected with either WT or Mut constructs alongside miR‐330‐5p mimics or a negative control miRNA (miR‐NC; RiboBio, China) using Lipofectamine 3000 (Invitrogen, USA) following the manufacturer's instructions. At 48 h post‐transfection, luciferase activity was measured using the Dual‐Luciferase Reporter Assay System (Promega, USA). Firefly luciferase activity was normalized to Renilla luciferase activity to control for transfection efficiency, and luminescence signals were recorded using a GloMax 20/20 luminometer (Promega, USA). A significant reduction in luciferase activity in the WT group upon miR‐330‐5p mimic transfection, but not in the Mut group, would confirm direct and specific binding of miR‐330‐5p to the TWIST1 3‐UTR.

### 2.7 Western Blot Analysis

Total protein was extracted from both cultured chondrocytes and cartilage tissue samples using RIPA lysis buffer (Sigma‐Aldrich, USA) supplemented with a protease inhibitor cocktail (Roche, Switzerland), and protein concentration was quantified using the BCA Protein Assay Kit (Thermo Fisher Scientific, USA). Equal amounts of protein (30 μg per lane) were resolved by SDS‐PAGE on 10% polyacrylamide gels and subsequently transferred onto PVDF membranes (Millipore, USA). Membranes were blocked with 5% non‐fat dried milk in TBST at room temperature for 1 h, then incubated overnight at 4°C with the following primary antibodies: anti‐TWIST1 (ab50887, Abcam, UK; 1:1000), anti‐Collagen I (COL1A1 (E8F4L) Rabbit Monoclonal Antibody #72026, Cell Signalling Technology, USA; 1:1000), anti‐Collagen II (ab34712, Abcam, UK; 1:1000), anti‐ADAMTS5 (ab41037, Abcam, UK; 1:1000), anti‐MMP3 (ab52915, Abcam, UK; 1:1000), anti‐MMP13 (ab39012, Abcam, UK; 1:1000), anti‐NF‐κB p65 (ab16502, Abcam, UK; 1:1000), anti‐phospho‐NF‐κB p65 (Ser536) (ab86299, Abcam, UK; 1:1000), and β‐actin (mouse monoclonal, ab8226, Abcam, UK; 1:5000). Following three washes with TBST, membranes were incubated with appropriate HRP‐conjugated secondary antibodies for 1 h at room temperature: goat anti‐rabbit IgG HRP (ab6721, Abcam, UK; 1:5000) for rabbit primary antibodies, and goat anti‐mouse IgG HRP (ab6789, Abcam, UK; 1:5000) for mouse primary antibodies. Protein bands were visualized by enhanced chemiluminescence using an ECL kit (Thermo Fisher Scientific, USA) and densitometric quantification was performed using ImageJ software (NIH, USA). All target protein expression levels were normalized to β‐actin.

### 2.8 Cell Viability Assay

Cell viability was assessed using the Cell Counting Kit‐8 (CCK‐8; Dojindo Molecular Technologies, Japan) according to the manufacturer's instructions. Briefly, chondrocytes were seeded in 96‐well plates at a density of 5 × 10^3^ cells per well and allowed to adhere overnight. Following the indicated treatments, cells were incubated for a further 48 h, after which 10 μL of CCK‐8 solution was added to each well and incubated at 37°C for 2 h. Absorbance was measured at 450 nm using a microplate reader (BioTek, USA). Cell viability was expressed as a percentage relative to the untreated control group, and all experiments were performed in triplicate.

### 2.9 5‐Ethynyl‐2’‐Deoxyuridine (EdU) Proliferation Assay

Cell proliferation was evaluated using the BeyoClick EdU‐594 Cell Proliferation Kit (Beyotime Biotechnology, China) according to the manufacturer's instructions. Briefly, chondrocytes were seeded in 24‐well plates at a density of 1 × 10^4^ cells per well and subjected to the indicated treatments. At 48 h post‐treatment, cells were incubated with 10 μM EdU labeling solution at 37°C for 2 h to allow incorporation of EdU into newly synthesized DNA. Cells were then fixed with 4% paraformaldehyde for 15 min at room temperature, permeabilized with 0.3% Triton X‐100 in PBS for 10 min, and subsequently incubated with the Click reaction cocktail containing Alexa Fluor 594‐azide for 30 min at room temperature in the dark. Cell nuclei were counterstained with DAPI (1 μg/mL) for 10 min. Images were acquired using a fluorescence microscope (Olympus, Japan), and EdU‐positive cells (red) were counted relative to total DAPI‐stained nuclei (blue) in at least five randomly selected fields per well.

### 2.10 Apoptosis Assay

Cell apoptosis was assessed by flow cytometry using the Annexin V‐FITC/Propidium Iodide (PI) Apoptosis Detection Kit (Beyotime Biotechnology, China) according to the manufacturer's instructions. Briefly, following 48 h of indicated treatments, chondrocytes were harvested by trypsinization, washed twice with ice‐cold PBS, and resuspended in 1× binding buffer at a density of 1 × 10^6^ cells/mL. Cells were then incubated with 5 μL Annexin V‐FITC and 5 μL PI for 15 min at room temperature in the dark. Apoptotic cells were immediately analysed using a flow cytometer (BD Biosciences, USA), with a minimum of 10,000 events recorded per sample. Data were analysed using FlowJo software (TreeStar, USA).

### 2.11 Enzyme‐Linked Immunosorbent Assay (ELISA)

The concentrations of TNF‐α, IL‐6, and IL‐8 were quantified in both cell culture supernatants and tissue homogenates using commercially available ELISA kits according to the manufacturer's instructions. For human samples, the following kits were used: Human TNF‐α DuoSet ELISA (DY210, R&D Systems, USA), Human IL‐6 DuoSet ELISA (DY206, R&D Systems, USA), and Human IL‐8/CXCL8 DuoSet ELISA (DY208, R&D Systems, USA). For mouse samples, the corresponding kits were used: Mouse TNF‐α DuoSet ELISA (DY410, R&D Systems, USA) and Mouse IL‐6 DuoSet ELISA (DY406, R&D Systems, USA). Cell culture supernatants were collected following 48 h of indicated treatments and centrifuged at 1000 × g for 10 min at 4°C to remove cellular debris prior to analysis. For tissue samples, cartilage tissues were homogenized in ice‐cold PBS supplemented with protease inhibitor cocktail (Roche, Switzerland), followed by centrifugation at 12,000 × g for 15 min at 4°C, and supernatants were collected for analysis. Protein concentrations of tissue homogenates were determined using the BCA Protein Assay Kit (Thermo Fisher Scientific, USA) to normalize cytokine concentrations. Absorbance was measured at 450 nm with wavelength correction at 570 nm using a microplate reader (BioTek, USA). Cytokine concentrations were calculated from standard curves and expressed as pg/mL or pg/mg protein. All samples were measured in triplicate.

### 2.12 Animal Model and Treatment

#### 2.12.1 Animal Model and In Vivo Experimental Design

All animal experiments were approved by the Animal Ethics Committee of Putuo Hospital, Shanghai University of Traditional Chinese Medicine, and conducted in strict accordance with the Basel Declaration and the ARRIVE guidelines 2.0 (https://arriveguidelines.org). Eight‐week‐old male C57BL/6J mice (body weight 22–25 g) were obtained from SPF (Beijing) Biotechnology Co. Ltd. and housed under specific pathogen‐free conditions in a temperature‐controlled facility (22°C ± 2°C, 50% ± 5% relative humidity) under a 12 h light/dark cycle, with ad libitum access to standard chow and sterilized water. After 1 week of acclimatization, mice were randomly assigned to four groups (*n* = 6 per group) using a random number table: Sham, DMM, DMM+Lv‐sh‐NC, and DMM+Lv‐sh‐circ_0003423.

#### 2.12.2 Surgical Procedure and Lentiviral Delivery

Osteoarthritis was surgically induced via destabilization of the medial meniscus (DMM) as previously described. Briefly, mice were anesthetised with an intraperitoneal injection of 1% pentobarbital sodium (50 mg/kg), and anaesthetic depth was confirmed by absence of pedal withdrawal reflex. A medial para‐patellar incision was made under aseptic conditions, the joint capsule was opened, and the medial menisco‐tibial ligament was carefully transected under a surgical microscope to destabilize the medial meniscus. In the Sham group, the ligament was exposed but left intact. The joint capsule and skin were closed in layers with absorbable sutures, and all mice received a single postoperative subcutaneous injection of penicillin (50,000 U/kg) to prevent infection. For intra‐articular gene silencing, lentivirus encoding shRNA targeting circ_0003423 (Lv‐sh‐circ_0003423) or a non‐targeting control shRNA (Lv‐sh‐NC; GenePharma, China) was administered at a titre of 1 × 10^8^ TU/mL in a 20 μL volume via intra‐articular injection into the right knee joint immediately following surgery using a 33‐gauge microsyringe under sterile conditions. Animals were monitored daily for signs of pain, infection, or abnormal behaviour throughout the experimental period.

#### 2.12.3 Tissue Collection and Analysis

At four weeks post‐surgery, all mice were euthanized by CO_2_ inhalation followed by cervical dislocation as a confirmatory method, in accordance with institutional guidelines. Knee joints were harvested bilaterally; one joint was processed for histological analysis and the contralateral joint cartilage was carefully dissected under a stereomicroscope for molecular analyses. For histological evaluation, knee joints were fixed in 10% neutral‐buffered formalin for 48 h, decalcified in 10% EDTA (pH 7.4) for 4 weeks at 4°C with solution changes every 3 days, and embedded in paraffin. Serial sections (5 μm thickness) were cut in the coronal plane through the medial compartment. Haematoxylin and Eosin (H&E) staining was performed using the H&E Staining Kit (Beyotime Biotechnology, China) to assess general tissue morphology, and Safranin O/Fast Green staining was performed using the Safranin O/Fast Green Staining Kit (Solarbio Science & Technology, China) to evaluate proteoglycan content and cartilage integrity. Histological severity of OA was graded using the Osteoarthritis Research Society International (OARSI) scoring system by two independent blinded observers. Chondrocyte apoptosis in tissue sections was assessed using the TUNEL Apoptosis Detection Kit (Beyotime Biotechnology, China) according to the manufacturer's instructions, and TUNEL‐positive cells were quantified as a percentage of total chondrocytes in at least five randomly selected fields per section.

### 2.13 Statistical Analysis

All Quantitative Data Were Derived From a Minimum of Three Independent Biological Replicates, Each Performed Using Cells Obtained From Three Separate Experimental Batches, Unless Otherwise Stated. For In Vivo Experiments, Data Were Obtained From Six Animals Per Group (*n* = 6). Technical Replicates (Repeated Measurements Within the Same Experiment) Were Averaged Prior to Statistical Analysis and Are Not Treated as Independent Observations. Data Are Expressed as Mean ± Standard Deviation (SD). Prior to Statistical Analysis, Normality of Data Distribution Was Assessed Using the Shapiro–Wilk Test, and Homogeneity of Variance Was Confirmed Using Levene's Test. All Datasets Satisfied the Assumption of Normality and Were Therefore Analysed Using Parametric Tests. Statistical Analyses Were Conducted Using GraphPad Prism 9.0 (GraphPad Software, USA). Differences Between Two Independent Groups Were Evaluated Using an Unpaired Two‐Tailed Student's *t*‐test. Comparisons Among Three or More Groups Were Assessed by One‐Way Analysis of Variance (ANOVA) Followed by Tukey's Post Hoc Test for Multiple Comparisons, Which Was Selected to Control for Family‐Wise Error Rate When Comparing All Group Pairs. For Histological OARSI Scoring, Which Represents Ordinal Data, Differences Between Groups Were Assessed Using the Kruskal‐Wallis Test Followed by Dunn's Post Hoc Test With Bonferroni Correction. A *p*‐value < 0.05 Was Considered Statistically Significant.

## 3 Results

### 3.1 Knockdown of Circ_0003423 Alleviates IL‐1β‐Induced Cell Viability Inhibition and Apoptosis in Chondrocytes

**FIGURE 1:**
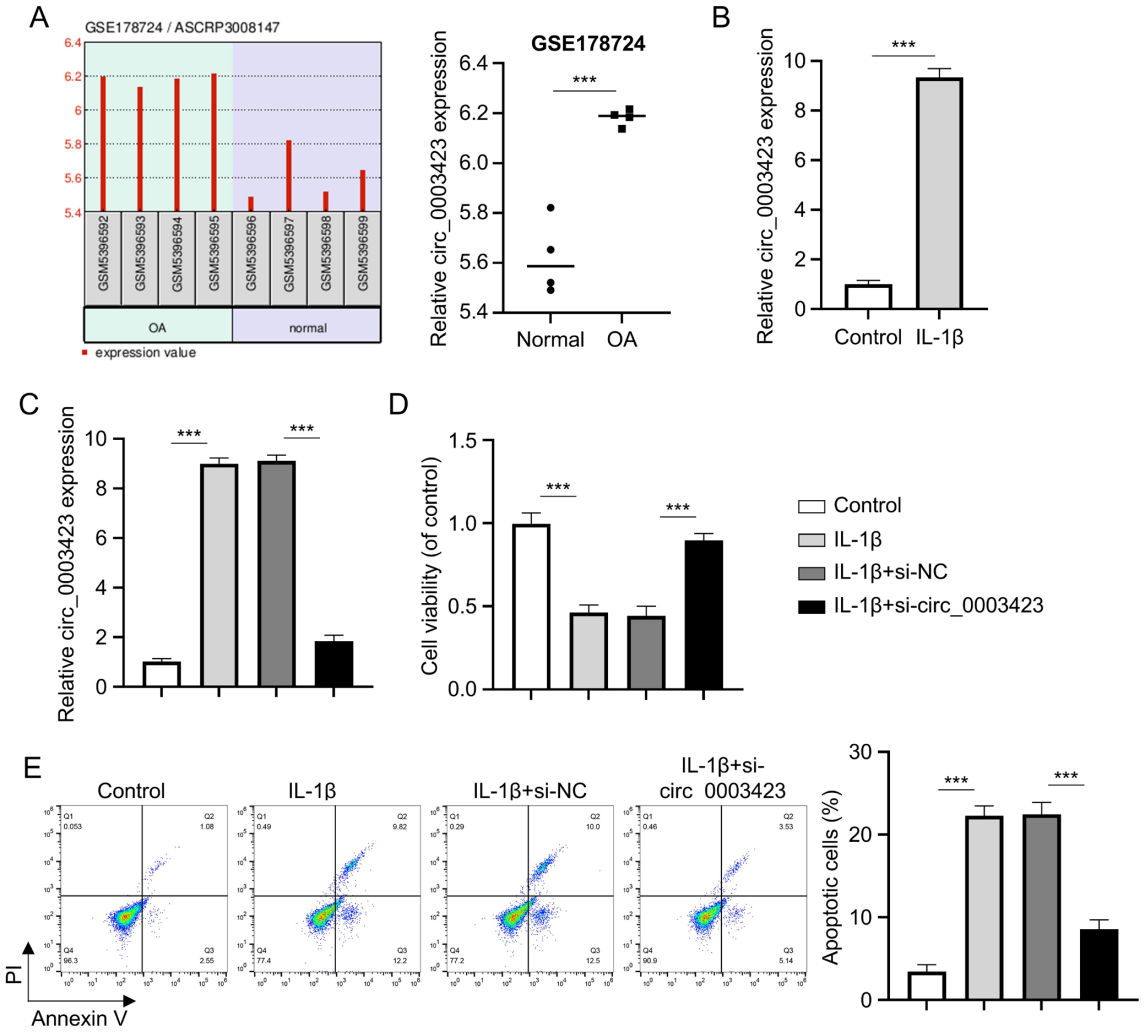
Knockdown of circ_0003423 alleviates IL‐1β‐induced inhibition of cell viability and promotion of apoptosis in chondrocytes. Human chondrocytes were treated with IL‐1β (10 ng/mL, 24 h) and transfected with si‐NC or si‐circ_0003423, generating the following groups: Control, IL‐1β, IL‐1β+si‐NC, and IL‐1β+si‐circ_0003423. (A) circ_0003423 expression levels in OA cartilage tissues relative to normal controls based on GSE178724 dataset analysis. (B) qRT‐PCR quantification of circ_0003423 expression in chondrocytes following IL‐1β stimulation. (C) qRT‐PCR assessment of si‐circ_0003423 knockdown efficiency in IL‐1β‐stimulated chondrocytes. (D) CCK‐8 assay evaluation of chondrocyte viability across experimental groups. (E) Flow cytometric analysis using Annexin V‐FITC/PI double staining to assess chondrocyte apoptosis. *N* = 3 independent experiments per group. ***p < 0.001.

Bioinformatic analysis of the publicly available GSE178724 dataset revealed that circ_0003423 (annotated as ASCRP3008147, hsa_circRNA_102378, hsa_circ_0003423, and mmu_circ_0000884) was significantly upregulated in osteoarthritic cartilage tissues compared with healthy controls (Figure 1A). To validate this finding at the cellular level, qRT‐PCR analysis demonstrated a marked and significant elevation of circ_0003423 expression in chondrocytes following 24 h of IL‐1β stimulation relative to untreated controls (Figure 1B), corroborating the clinical dataset observations. To confirm the circular nature of circ_0003423, three complementary validation approaches were employed (Figure S1). First, PCR amplification using divergent primers yielded a distinct amplification product from cDNA but produced no detectable band from genomic DNA (gDNA), whereas convergent primers generated the expected amplification products from both templates, confirming the existence of a bona fide back‐splice junction in circ_0003423. Second, PCR amplification using both divergent and convergent primers yielded clearly visible bands in RNase R‐treated cDNA, indicating that circ_0003423 resisted RNase R‐mediated linear RNA degradation (Figure S1). Third, Sanger sequencing of the divergent primer‐amplified PCR product unambiguously confirmed the identity of the back‐splice junction, with the resultant sequence precisely mapped to the corresponding genomic locus upon alignment to the reference genome (Figure S1). Collectively, these three lines of evidence validate the circular structure of circ_0003423 and confirm the authenticity of its back‐splice junction.

To investigate the functional role of circ_0003423, small interfering RNA‐mediated knockdown (si‐circ_0003423) was employed, which effectively and significantly attenuated the IL‐1β‐induced upregulation of circ_0003423 expression (Figure 1C), confirming successful gene silencing. Subsequent functional assessments revealed that IL‐1β treatment significantly reduced chondrocyte cell viability, as evidenced by CCK‐8 assay; notably, si‐circ_0003423 co‐transfection substantially restored cell viability to levels approaching those of untreated cells (Figure 1D). Furthermore, flow cytometric analysis using Annexin V‐FITC/PI double staining demonstrated that IL‐1β stimulation markedly promoted chondrocyte apoptosis, whereas knockdown of circ_0003423 via si‐circ_0003423 co‐transfection significantly largely counteracted this pro‐apoptotic effect (Figure 1E). Collectively, these findings provide evidence that circ_0003423 is pathologically upregulated under inflammatory conditions, and that its silencing effectively restores cell viability and mitigates IL‐1β‐induced apoptosis in chondrocytes, suggesting a potentially critical regulatory role for circ_0003423 in OA chondrocyte pathology.

### 3.2 Knockdown of circ_0003423 Alleviates IL‐1β‐Induced Inflammation and ECM Degradation in Chondrocytes

**FIGURE 2:**
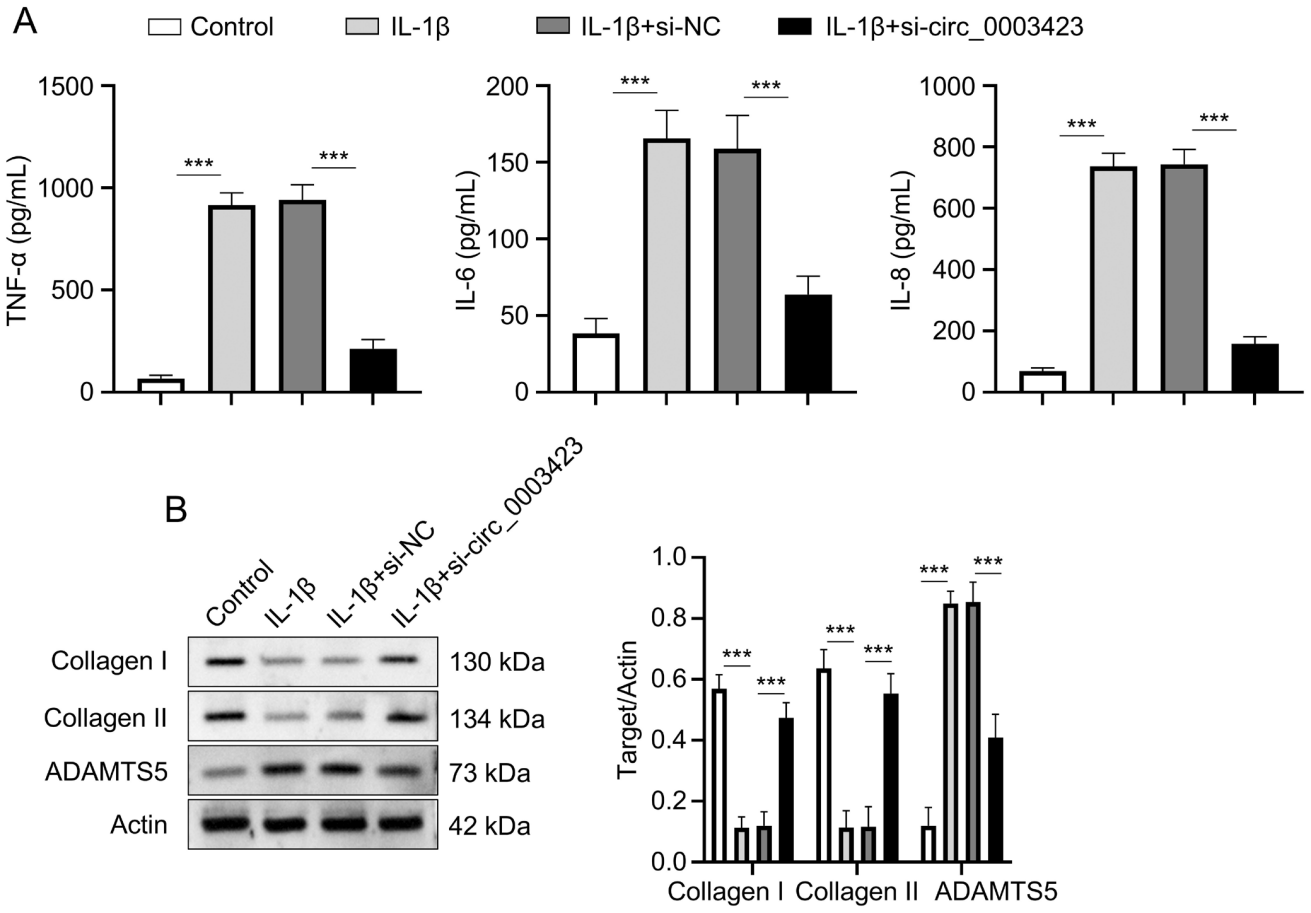
Knockdown of circ_0003423 mitigates IL‐1β‐induced inflammation and extracellular matrix degradation in chondrocytes. Human chondrocytes were treated with IL‐1β (10 ng/mL, 24 h) and transfected with si‐NC or si‐circ_0003423, generating the following groups: Control, IL‐1β, IL‐1β+si‐NC, and IL‐1β+si‐circ_0003423. (A) ELISA quantification of pro‐inflammatory cytokines TNF‐α, IL‐6, and IL‐8 in cell culture supernatants across experimental groups. (B) Western blot analysis of ECM‐related proteins including Collagen I, Collagen II, and ADAMTS5 across experimental groups. *N* = 3 independent experiments per group. ***p < 0.001.

We next evaluated the regulatory role of circ_0003423 in IL‐1β‐driven inflammatory responses and extracellular matrix (ECM) homeostasis. ELISA quantification of culture supernatants demonstrated that IL‐1β stimulation markedly and significantly elevated the secretion of pro‐inflammatory cytokines, including IL‐6, TNF‐α, and IL‐8, compared with untreated controls; importantly, si‐circ_0003423 co‐transfection substantially attenuated the IL‐1β‐induced elevation of all three cytokines (Figure 2A). With respect to ECM metabolism, Western blot analysis revealed that IL‐1β stimulation significantly upregulated the expression of the matrix‐degrading enzyme ADAMTS5 while concurrently downregulating the structural matrix proteins Collagen II and Collagen I, reflecting a shift toward a catabolic ECM state. Notably, knockdown of circ_0003423 via si‐circ_0003423 effectively reversed these IL‐1β‐induced alterations, partially restoring the expression of Collagen I and Collagen II while suppressing ADAMTS5 upregulation (Figure 2B). Taken together, these results demonstrate that silencing circ_0003423 exerts protective effects in IL‐1β‐stimulated chondrocytes by simultaneously dampening pro‐inflammatory cytokine release and preserving ECM integrity.

### 3.3 circ_0003423 Directly Targets and Sponges miR‐330‐5p in Chondrocytes

**FIGURE 3:**
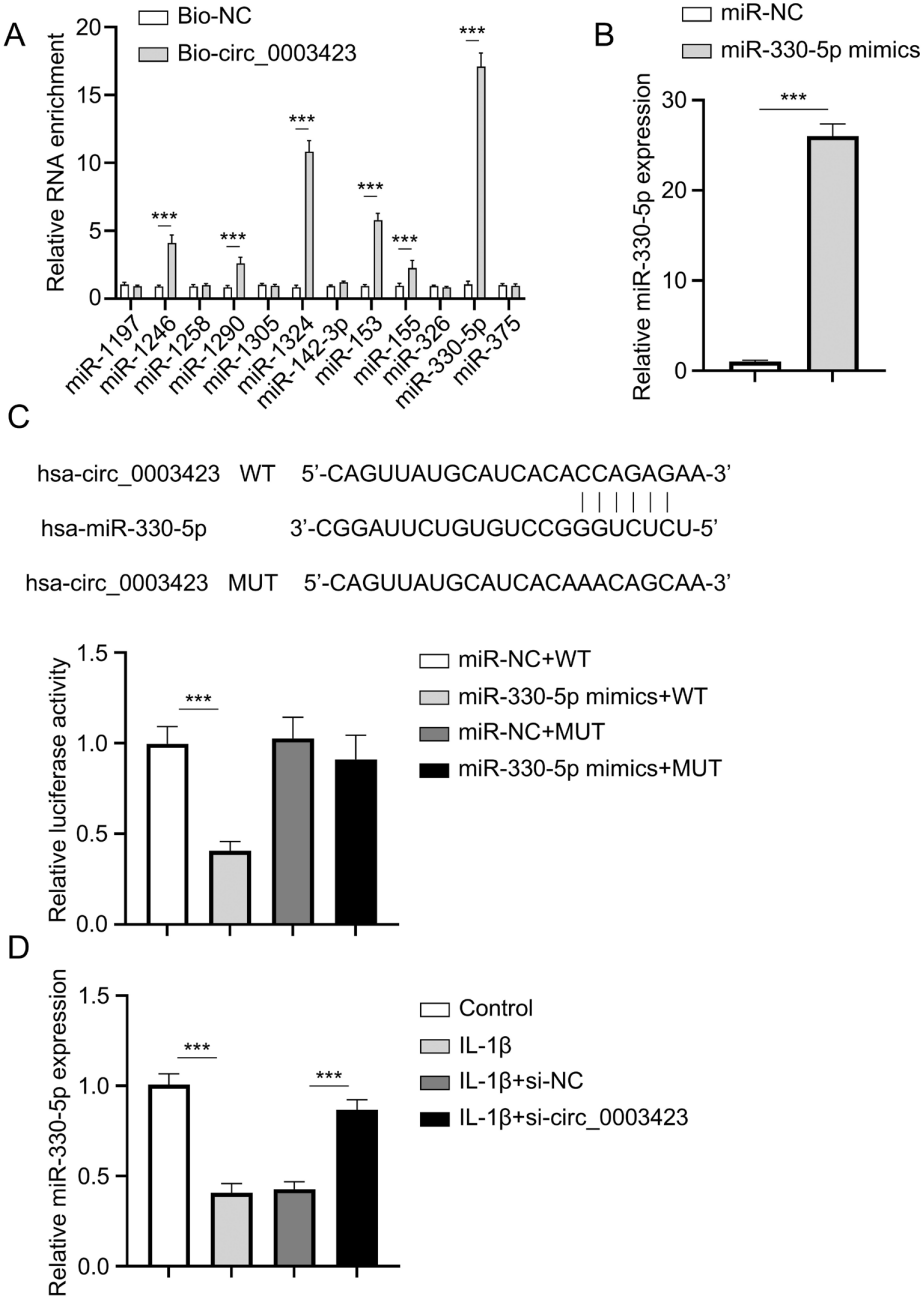
Circ_0003423 directly sponges miR‐330‐5p in chondrocytes. To investigate the interaction between circ_0003423 and miR‐330‐5p, chondrocytes were subjected to RNA pull‐down, luciferase reporter assay, and molecular expression analyses under IL‐1β stimulation and circ_0003423 knockdown conditions. (A) RNA pull‐down assay using a biotin‐labelled circ_0003423 probe to assess the enrichment of miR‐330‐5p. (B) qRT‐PCR verification of miR‐330‐5p overexpression efficiency following mimic transfection. (C) Dual‐luciferase reporter assay assessing the binding specificity between circ_0003423 and miR‐330‐5p using wild‐type and mutant constructs co‐transfected with miR‐330‐5p mimic or miR‐NC. (D) qRT‐PCR analysis of miR‐330‐5p expression levels in chondrocytes following IL‐1β stimulation and/or circ_0003423 knockdown. *N* = 3 independent experiments per group. ***p < 0.001.

We next sought to identify potential miRNA targets of circ_0003423; bioinformatic analysis using circBase database predicted 12 candidate miRNAs harbouring putative binding sites for circ_0003423. RNA pull‐down assay showed the strongest specific enrichment of miR‐330‐5p in the circ_0003423 pull‐down fraction compared with the control group, providing initial biochemical evidence of an interaction between circ_0003423 and miR‐330‐5p (Figure 3A). Successful and efficient overexpression of miR‐330‐5p was subsequently confirmed by qRT‐PCR following mimic transfection, validating the transfection system for downstream functional assays (Figure 3B). To verify the functional interaction, dual‐luciferase reporter assays demonstrated that miR‐330‐5p mimic transfection significantly suppressed luciferase activity of reporter constructs carrying the wild‐type circ_0003423 binding sequence, whereas luciferase activity of the corresponding mutant constructs remained unaffected, suggesting the specificity of circ_0003423–miR‐330‐5p interaction (Figure 3C). Furthermore, RNA immunoprecipitation (RIP) assay using an anti‐Ago2 antibody demonstrated significant co‐enrichment of both circ_0003423 and miR‐330‐5p in the Ago2 immunoprecipitate compared with the IgG control group, indicating that both molecules are incorporated into the same RNA‐induced silencing complex (RISC) and confirming their functional interaction within the endogenous miRNA machinery (Figure S2). Additionally, qRT‐PCR analysis revealed that IL‐1β stimulation significantly suppressed endogenous miR‐330‐5p expression, whereas knockdown of circ_0003423 partially but significantly restored miR‐330‐5p levels (Figure 3D). Collectively, these data provide evidence that circ_0003423 directly interacts with and sequesters miR‐330‐5p through a specific binding site in chondrocytes.

### 3.4 miR‐330‐5p Directly Targets and Negatively Regulates TWIST1 in Chondrocytes

**FIGURE 4:**
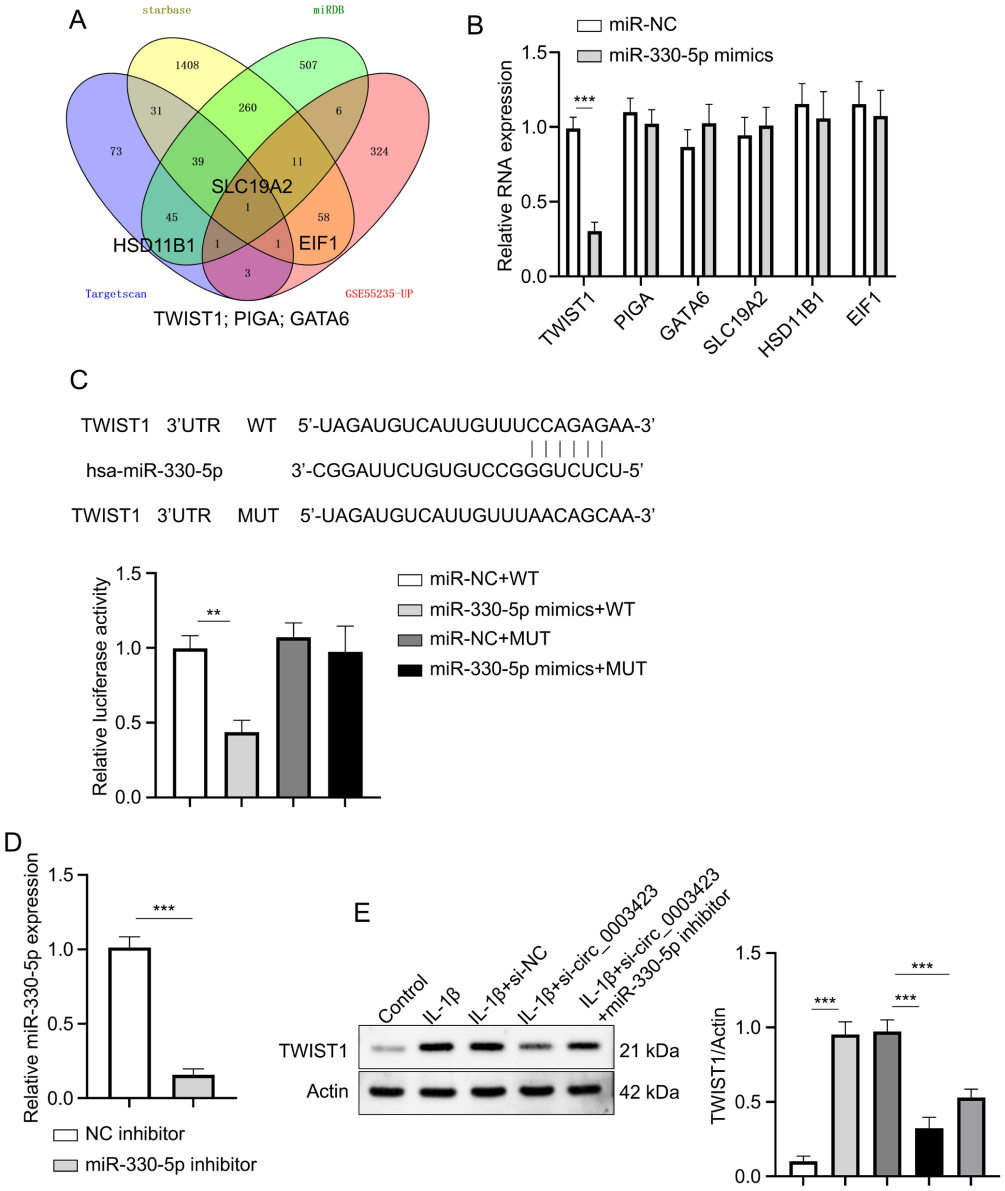
miR‐330‐5p directly targets and negatively regulates TWIST1 in chondrocytes. To identify downstream targets of miR‐330‐5p and validate the miR‐330‐5p/TWIST1 interaction, bioinformatic analysis, luciferase reporter assays, and molecular expression analyses were performed in chondrocytes transfected with miR‐330‐5p mimics or inhibitors under IL‐1β stimulation and circ_0003423 knockdown conditions. (A) Venn diagram illustrating the overlap of miR‐330‐5p candidate target genes predicted by StarBase, miRDB, and TargetScan databases with differentially expressed genes from the GSE55235 dataset. (B) QRT‐PCR evaluation of candidate target gene expression levels following miR‐330‐5p mimic overexpression. (C) Dual‐luciferase reporter assay validating the direct binding between miR‐330‐5p and the wild‐type or mutant TWIST1 3′‐UTR. (D) qRT‐PCR confirmation of miR‐330‐5p inhibitor transfection efficiency. (E) Western blot analysis of TWIST1 protein expression in chondrocytes subjected to IL‐1β stimulation, circ_0003423 knockdown, and/or miR‐330‐5p inhibition. *N* = 3 independent experiments per group. **p < 0.01; ***p < 0.001.

To identify downstream targets of miR‐330‐5p, an integrated bioinformatic approach was employed combining three independent target prediction databases, StarBase, miRDB, and TargetScan, with differentially expressed genes in the GSE55235 dataset. This analysis converged on six candidate target genes, namely TWIST1, PIGA, GATA6, SLC19A2, HSD11B1, and EIF1 (Figure 4A). Subsequent qRT‐PCR analysis demonstrated that overexpression of miR‐330‐5p via mimic transfection specifically and significantly reduced TWIST1 mRNA expression levels, suggesting TWIST1 as a primary downstream target of miR‐330‐5p in chondrocytes (Figure 4B). The direct interaction between miR‐330‐5p and the TWIST1 3′‐UTR was further confirmed by dual‐luciferase reporter assays, in which miR‐330‐5p mimic transfection significantly suppressed luciferase activity in cells harbouring the wild‐type TWIST1 3′‐UTR construct, whereas no significant alteration was observed in cells transfected with the mutant construct (Figure 4C). Efficient suppression of endogenous miR‐330‐5p expression was subsequently confirmed by qRT‐PCR following transfection with miR‐330‐5p inhibitors (Figure 4D). At the protein level, Western blot analysis revealed that IL‐1β stimulation markedly increased TWIST1 protein expression, which was significantly attenuated by circ_0003423 knockdown; notably, co‐treatment with the miR‐330‐5p inhibitor rescued TWIST1 protein levels, reversing the suppressive effect of circ_0003423 silencing (Figure 4E). Collectively, these findings establish that miR‐330‐5p targets and negatively regulates TWIST1 expression in chondrocytes through a specific 3′‐UTR binding interaction.

### 3.5 The circ_0003423/miR‐330‐5p/TWIST1 Axis Regulates IL‐1β‐Induced Cell Death and ECM Changes in Chondrocytes

**FIGURE 5:**
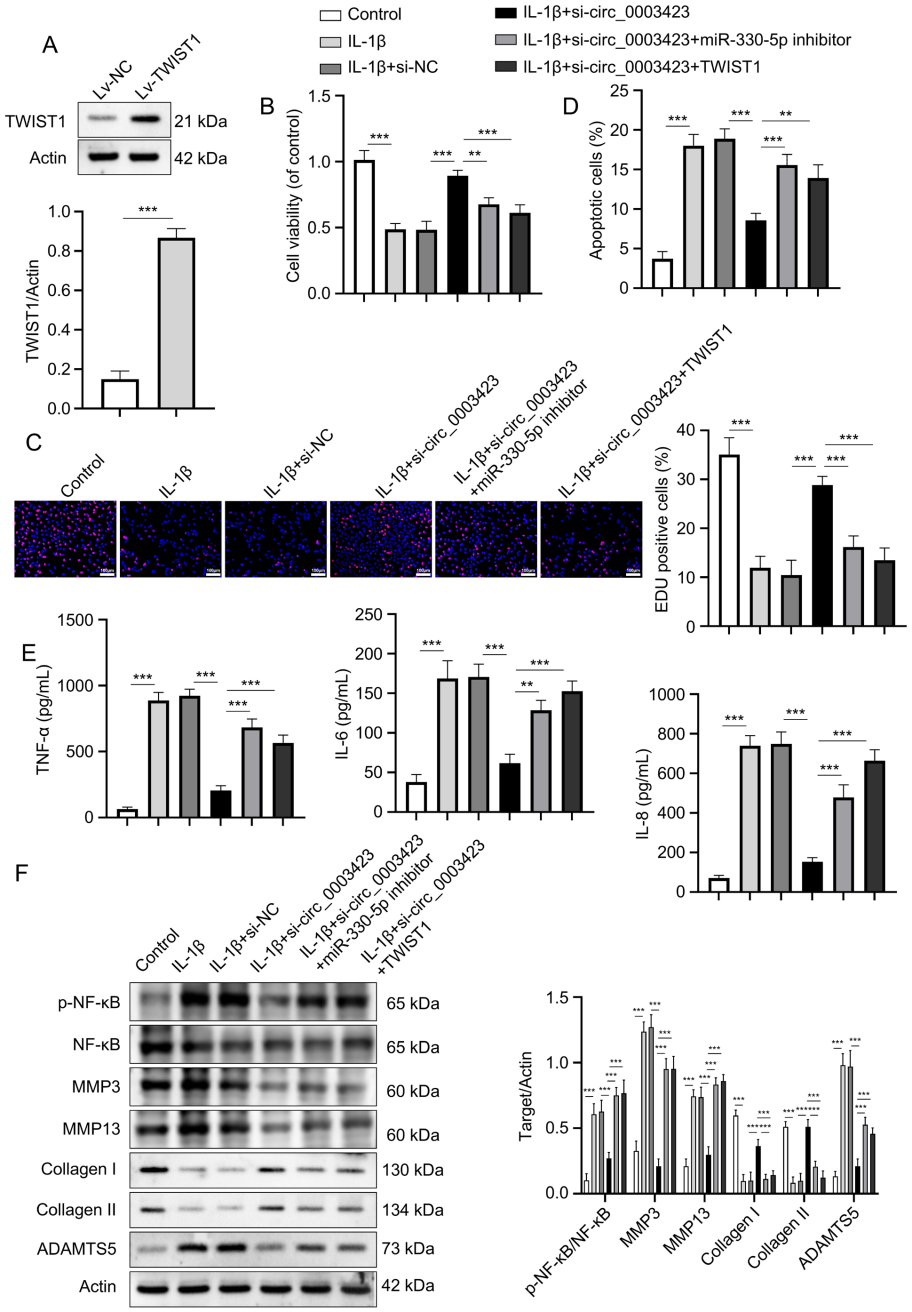
The circ_0003423/miR‐330‐5p/TWIST1 axis regulates IL‐1β‐induced chondrocyte injury. To validate the functional role of the circ_0003423/miR‐330‐5p/TWIST1 regulatory axis, rescue experiments were performed in human chondrocytes under six treatment conditions: Control, IL‐1β, IL‐1β+si‐NC, IL‐1β+si‐circ_0003423, IL‐1β+si‐circ_0003423+miR‐330‐5p inhibitor, and IL‐1β+si‐circ_0003423+TWIST1 overexpression. (A) Western blot confirmation of TWIST1 overexpression efficiency across treatment groups. (B) CCK‐8 assay evaluation of chondrocyte viability across experimental groups. (C) EdU incorporation assay assessment of chondrocyte proliferation across experimental groups. (D) Flow cytometric analysis using Annexin V‐FITC/PI double staining to evaluate chondrocyte apoptosis. (E) ELISA quantification of pro‐inflammatory cytokine levels in cell culture supernatants across experimental groups. (F) Western blot analysis of Collagen I, Collagen II, MMP3, MMP13, ADAMTS5, total NF‐κB p65, and phosphorylated NF‐κB p65 expression across experimental groups. *N* = 3 independent experiments per group. **p < 0.01; ***p < 0.001.

To functionally validate the circ_0003423/miR‐330‐5p/TWIST1 regulatory axis, chondrocytes were treated under six conditions: Control, IL‐1β, IL‐1β+si‐NC, IL‐1β+si‐circ_0003423, IL‐1β+si‐circ_0003423+miR‐330‐5p inhibitor, and IL‐1β+si‐circ_0003423+TWIST1. TWIST overexpression was confirmed by Western blot after transfection (Figure 5A). CCK‐8 assay revealed that IL‐1β significantly reduced chondrocyte viability, which was restored by circ_0003423 knockdown; however, this protective effect was reversed by either miR‐330‐5p inhibition or TWIST1 overexpression (Figure 5B). Consistently, EdU assay demonstrated that circ_0003423 knockdown significantly rescued IL‐1β‐induced suppression of chondrocyte proliferation, an effect that was similarly abolished upon miR‐330‐5p inhibition or TWIST1 restoration (Figure 5C). IL‐1β treatment also significantly promoted chondrocyte apoptosis, while circ_0003423 knockdown attenuated this effect, which was reversed by miR‐330‐5p inhibitor or TWIST1 overexpression (Figure 5D). Furthermore, ELISA analysis demonstrated that IL‐1β‐induced upregulation of pro‐inflammatory cytokine levels was significantly attenuated by circ_0003423 knockdown, whereas this suppressive effect was counteracted by miR‐330‐5p inhibition or TWIST1 overexpression (Figure 5E). At the protein level, Western blot analysis demonstrated that IL‐1β stimulation markedly downregulated Collagen I and Collagen II expression, and circ_0003423 knockdown significantly restored these matrix proteins; however, this rescue effect was abolished upon miR‐330‐5p inhibition or TWIST1 overexpression (Figure 5F). Concurrently, IL‐1β‐induced upregulation of matrix‐degrading enzymes, including MMP3, MMP13, and ADAMTS5, was significantly suppressed by circ_0003423 knockdown, whereas miR‐330‐5p inhibition or TWIST1 overexpression reversed this suppressive effect (Figure 5F). Furthermore, circ_0003423 knockdown significantly attenuated IL‐1β‐induced NF‐κB p65 phosphorylation; notably, this inhibitory effect on NF‐κB pathway activation was similarly counteracted by miR‐330‐5p inhibition or TWIST1 overexpression (Figure 5F). Collectively, these findings demonstrate that circ_0003423 drives IL‐1β‐induced chondrocyte injury through the miR‐330‐5p/TWIST1/NF‐κB axis.

### 3.6 Knockdown of circ_0003423 Alleviates Cartilage Injury in a Mouse Model of Osteoarthritis

**FIGURE 6:**
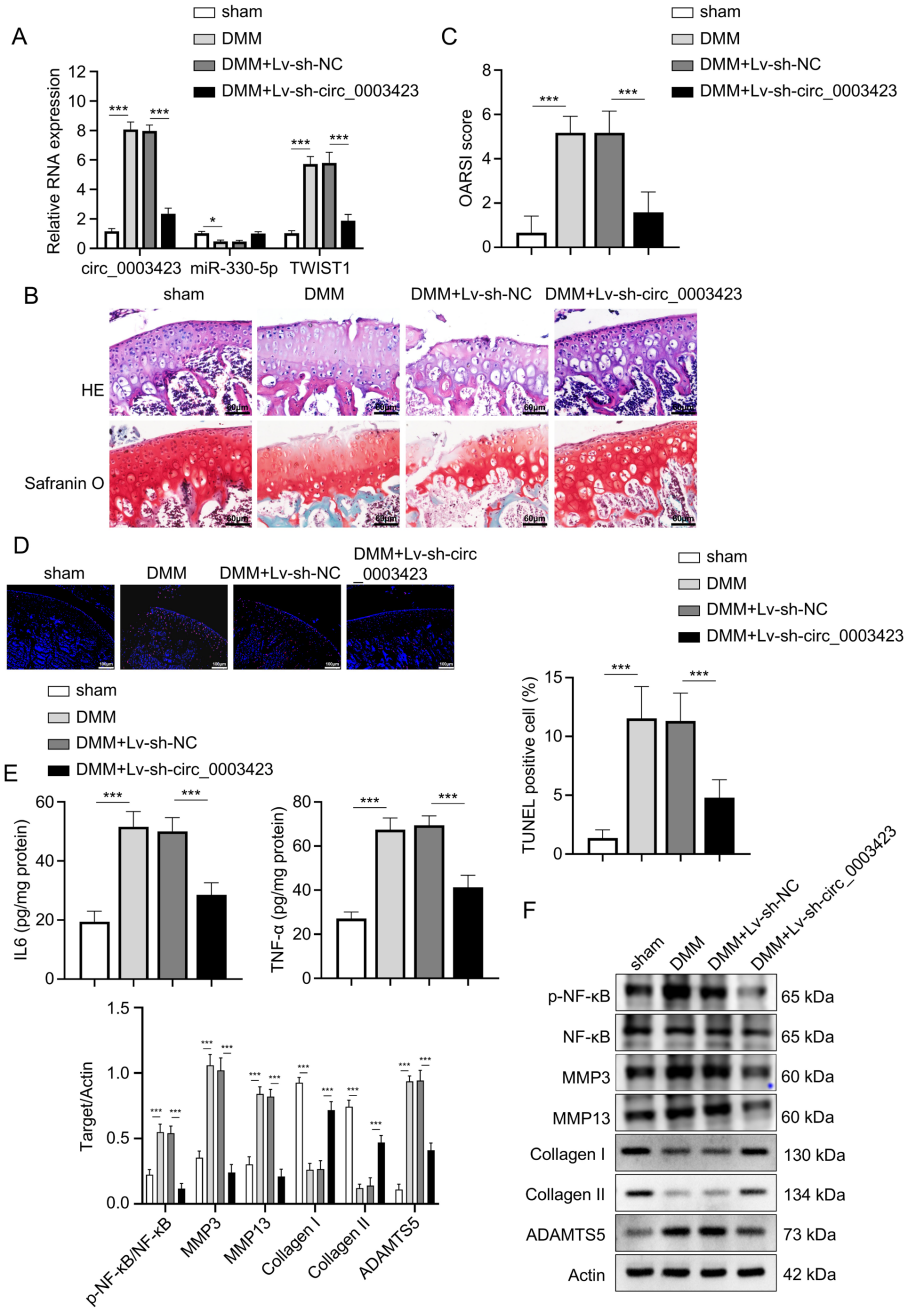
Knockdown of circ_0003423 alleviates cartilage injury in a mouse model of osteoarthritis. OA was surgically induced via DMM in C57BL/6J mice, followed by intra‐articular injection of circ_0003423 shRNA lentivirus or non‐targeting control lentivirus, generating the following groups: Sham, DMM, DMM+Lv‐sh‐NC, and DMM+Lv‐sh‐circ_0003423. (A) qRT‐PCR quantification of circ_0003423, miR‐330‐5p, and TWIST1 expression levels in mouse articular cartilage tissues. (B) Representative histological images of knee joint sections stained with Haematoxylin and Eosin and Safranin O/Fast Green to assess cartilage morphology and proteoglycan content. (C) Quantitative OARSI scoring of cartilage damage severity evaluated by two independent blinded observers. (D) TUNEL assay assessment of chondrocyte apoptosis in articular cartilage tissue sections. (E) ELISA measurement of IL‐6 and TNF‐α concentrations in articular cartilage tissue homogenates. (F) Western blot analysis of Collagen I, Collagen II, ADAMTS5, MMP3, MMP13, total NF‐κB p65, and phosphorylated NF‐κB p65 expression in cartilage tissue. *N* = 6 animals per group. *p < 0.05; ***p < 0.001.

To validate these findings In vivo, OA was induced in mice via DMM surgery, followed by intra‐articular injection of circ_0003423 shRNA lentivirus. QRT‐PCR confirmed that DMM surgery significantly increased circ_0003423 and TWIST1 expression while reducing miR‐330‐5p levels, and circ_0003423 knockdown partially reversed these changes (Figure 6A). Histological analysis by HE and Safranin O staining revealed marked cartilage destruction and proteoglycan loss in DMM mice, both of which were substantially alleviated by circ_0003423 knockdown (Figure 6B). OARSI scoring confirmed significantly reduced cartilage damage in the circ_0003423‐silenced group (Figure 6C). TUNEL assay indicated that chondrocyte apoptosis was markedly elevated following DMM surgery but was significantly attenuated after circ_0003423 silencing (Figure 6D). ELISA revealed that circ_0003423 knockdown significantly reduced the elevation of IL‐6 and TNF‐α in the joint cartilage tissues of DMM mice (Figure 6E). Western blot analysis of cartilage tissue further confirmed that DMM surgery markedly decreased Collagen I and Collagen II expression while upregulating ADAMTS5, MMP3, and MMP13 levels and enhancing NF‐κB p65 phosphorylation, all of which were significantly reversed by circ_0003423 knockdown (Figure 6F). Collectively, these findings suggest that circ_0003423 silencing ameliorates OA progression and suppresses NF‐κB‐mediated inflammation, reducing matrix degradation and protecting cartilage integrity In vivo.

## 4 Discussion

The present study identifies circ_0003423 as a pathologically upregulated circRNA in OA cartilage and IL‐1β‐stimulated chondrocytes, and systematically demonstrates that it drives OA progression by functioning as a ceRNA to sequester miR‐330‐5p, thereby de‐repressing TWIST1 and activating downstream NF‐κB signalling and MMP3/MMP13/ADAMTS5‐mediated ECM destruction. In vitro, circ_0003423 silencing rescued IL‐1β‐induced inhibition of chondrocyte viability and proliferation, attenuated apoptosis, suppressed pro‐inflammatory cytokine secretion, and restored ECM homeostasis, effects that were consistently abrogated by miR‐330‐5p inhibition or TWIST1 overexpression. In vivo validation in the DMM mouse model further confirmed that circ_0003423 knockdown upregulated miR‐330‐5p, suppressed TWIST1/NF‐κB/MMP signalling, and ameliorated cartilage degeneration, chondrocyte apoptosis, and joint inflammation. Collectively, these findings establish the circ_0003423/miR‐330‐5p/TWIST1/NF‐κB axis as a mechanistically coherent regulatory network in OA pathogenesis, and position circ_0003423 as a promising candidate therapeutic target.

A central finding of this study is that circ_0003423 regulates TWIST1 expression through miR‐330‐5p sponging in the context of IL‐1β‐driven chondrocyte injury. TWIST1 is a pleiotropic basic helix–loop–helix transcription factor that has been increasingly recognized as a critical mediator of inflammatory signalling and ECM catabolism in chondrocytes, with its aberrant upregulation directly linked to OA pathological progression [18]. Accordingly, miR‐330‐5p, which is consistently suppressed under inflammatory conditions, functions as a post‐transcriptional brake on TWIST1 expression, and its downregulation in OA effectively licences TWIST1‐driven cartilage destruction. The IL‐1β‐induced chondrocyte injury model employed in this study is well‐established and recapitulates key pathological hallmarks of OA at the cellular level, including inflammatory cytokine release, apoptosis, and ECM imbalance [19], thereby providing a physiologically relevant platform to interrogate the functional contribution of circ_0003423. Our data demonstrate that circ_0003423 knockdown substantially reversed these IL‐1β‐induced cellular insults, implicating this circRNA as an upstream regulator of the inflammatory and catabolic cascade in chondrocytes. These observations are consistent with the well‐documented roles of NF‐κB pathway hyperactivation [20] and dysregulated miRNA‐mediated gene networks [21] as central drivers of chondrocyte dysfunction in OA, and extend this framework by placing circ_0003423 as an upstream epigenetic amplifier of these pathological processes.

The regulatory paradigm uncovered in this study, whereby circ_0003423 acts as a miRNA sponge to modulate a disease‐relevant mRNA target, is increasingly recognized as a conserved and functionally significant mechanism across multiple OA‐associated circRNAs [22, 23, 24]. Comprehensive bioinformatic and experimental studies have catalogued numerous circRNA–miRNA–mRNA regulatory networks that govern chondrocyte fate and cartilage integrity, underscoring the breadth of circRNA involvement in OA pathobiology. A particularly instructive parallel is provided by circSOD2, which promotes OA progression by sequestering miR‐224‐5p and thereby de‐repressing its target PRDX3, ultimately exacerbating oxidative stress‐related chondrocyte injury [25]. Much like circ_0003423, circSOD2 exemplifies how individual circRNAs can hijack specific miRNA–mRNA regulatory nodes to amplify pathological signalling cascades in chondrocytes [26]. Taken together, these findings reinforce the concept that circRNA‐mediated ceRNA networks constitute a common but mechanistically distinct layer of epigenetic regulation in OA, with each circRNA engaging unique downstream effectors to orchestrate disease progression.

Among the downstream effectors of the circ_0003423/miR‐330‐5p axis, TWIST1 occupies a particularly pivotal position. Prior studies have established that TWIST1 promotes cartilage catabolism in OA through transcriptional upregulation of MMP3, a process mechanistically linked to TWIST1‐induced alterations in DNA hydroxymethylation at the MMP3 promoter [27]. Our data extend this mechanistic understanding by demonstrating that circ_0003423‐driven upregulation of TWIST1 not only elevates MMP3 but also augments MMP13 expression and activates NF‐κB p65 phosphorylation, collectively amplifying the pro‐inflammatory and pro‐catabolic milieu in chondrocytes. Furthermore, the concomitant suppression of Collagen I and Collagen II synthesis under circ_0003423 overexpression conditions illustrates how this regulatory axis simultaneously accelerates matrix degradation and impairs matrix synthesis, creating a compounding catabolic state that is characteristic of advanced OA. The rescue of these molecular perturbations upon circ_0003423 silencing, and their re‐emergence following miR‐330‐5p inhibition or TWIST1 restoration, provides compelling mechanistic evidence that circ_0003423 acts upstream of TWIST1 through a miR‐330‐5p‐dependent mechanism to coordinate inflammatory signalling and ECM destruction in chondrocytes. Furthermore, the fact that TWIST1 lies at the nexus of miR‐330‐5p regulation and NF‐κB pathway activation suggests that it may integrate multiple upstream signalls to amplify cartilage damage, warranting further investigation of its broader transcriptional target repertoire beyond MMP3 and MMP13 [17].

From a translational perspective, the circ_0003423/miR‐330‐5p/TWIST1 axis represents a potentially actionable therapeutic target. The circular topology of circ_0003423 renders it highly resistant to exonucleolytic degradation, conferring exceptional molecular stability and making it an attractive candidate for RNA‐based silencing strategies. Approaches including circRNA‐specific small interfering RNAs and antisense oligonucleotides (ASOs) have demonstrated technical feasibility for targeting circRNAs [28], and circRNA‐directed RNA interference platforms have shown promising preclinical efficacy in cancer and neurological disease models [29, 30], providing proof‐of‐concept for their broader applicability in chronic inflammatory conditions such as OA. Complementarily, therapeutic restoration of miR‐330‐5p activity using synthetic mimics represents an alternative strategy; miR‐330‐5p has been demonstrated to modulate disease‐relevant downstream targets in diverse pathological contexts, including non‐small cell lung cancer and myocardial ischemia–reperfusion injury [31, 32], suggesting that its therapeutic restoration may exert pleiotropic protective effects beyond TWIST1 suppression in OA. Nevertheless, substantial translational barriers remain, including achieving joint‐specific delivery, ensuring adequate stability under inflammatory synovial conditions, minimizing off‐target effects, and characterizing potential immunostimulatory responses, all of which must be rigorously addressed before clinical application can be considered.

Several limitations of the present study warrant acknowledgment. Although robust In vitro and In vivo evidence collectively supports the oncogenic‐like role of circ_0003423 in OA, clinical validation in well‐powered human cohorts encompassing diverse disease stages is indispensable before any conclusions regarding its diagnostic or prognostic utility can be drawn. While this study delineates the circ_0003423/miR‐330‐5p/TWIST1/NF‐κB axis with considerable mechanistic resolution, TWIST1 is a multifunctional transcription factor with a broad target repertoire, and additional downstream signalling pathways through which TWIST1 may contribute to OA pathogenesis beyond those examined here remain to be systematically explored. Integrative transcriptomic and proteomic profiling approaches will be essential for comprehensively mapping these networks. Moreover, the upstream mechanisms responsible for circ_0003423 upregulation in OA are also currently undefined. While inflammatory mediators such as IL‐1β may drive its expression through epigenetic or transcriptional mechanisms, the contributions of mechanical loading, oxidative stress, and aging‐associated epigenetic remodelling merit dedicated investigation. Finally, the long‐term In vivo efficacy and safety profile of sustained circ_0003423 silencing remain to be established, particularly with regard to potential off‐target effects on non‐cartilaginous joint tissues.

## 5 Conclusion

In summary, our data suggest that circ_0003423 is pathologically upregulated in OA and drives disease progression by functioning as a ceRNA to sponge miR‐330‐5p, thereby de‐repressing TWIST1 and activating downstream NF‐κB signalling and MMP‐mediated ECM destruction in chondrocytes. Both In vitro and In vivo findings demonstrated that circ_0003423 silencing restored chondrocyte viability, attenuated apoptosis and inflammation, and preserved cartilage integrity, confirming the biological significance of the circ_0003423/miR‐330‐5p/TWIST1/NF‐κB regulatory axis in OA pathogenesis. These findings position circ_0003423 as a promising therapeutic target, and further clinical validation alongside the development of joint‐targeted RNA delivery strategies will be essential to translate these preclinical findings into effective therapeutic applications for OA.

## Author Contributions

**Lei Zhang:** conceptualization, investigation, writing – original draft, software, formal analysis, methodology, data curation. **Hua Wang:** data curation, software, methodology, investigation. **Shaoyang Liu:** investigation, methodology, formal analysis. **Fei Wu:** conceptualization, writing – review and editing, funding acquisition, project administration. **Jianjun Qiu:** formal analysis, methodology. **Yue Ding:** writing – review and editing, investigation, supervision.

## Funding

This study was supported by the Research Fund of Outstanding Discipline Backbone Talents (Rising Sun Project) at Shanghai Putuo People's Hospital (2025XR03) and Characteristic Special Disease Project: Degenerative Spinal Diseases (2024tszb06).

## Ethics Statement

Each animal experimental procedure gained approval from the Animal Ethics Committee of Putuo Hospital, Shanghai University of Traditional Chinese Medicine. The experimental protocol was performed in accordance with the relevant guidelines and regulations of the Basel Declaration. The study is reported in accordance with ARRIVE guidelines (https://arriveguidelines.org).

## Consent

The authors have nothing to report.

## Conflicts of Interest

The authors declare no conflicts of interest.

## Supporting information


**Figure S1:** Validation of the circular structure of circ_0003423. Complementary approaches were employed to confirm the circular nature of circ_0003423 in human chondrocytes: 1. PCR amplification using divergent and convergent primers with CDNA and genomic DNA (GDNA) as templates, with products resolved by agarose gel electrophoresis to confirm the existence of the back‐splice junction; 2. RNase R resistance assay in which total RNA was treated with or without RNase R, followed by PCR amplification using divergent and convergent primers to confirm resistance of circ_0003423 to linear RNA degradation; 3. Sanger sequencing chromatogram of the divergent primer‐amplified PCR product confirming the identity of the back‐splice junction of circ_0003423 upon alignment to the reference genome.

**Figure S2:** RNA immunoprecipitation (RIP) assay confirms co‐enrichment of circ_0003423 and miR‐330‐5p within the RISC complex. Anti‐Ago2 RIP assay was performed in human chondrocytes using an anti‐Ago2 antibody or normal rabbit IgG as a negative control. QRT‐PCR quantification of circ_0003423 and miR‐330‐5p enrichment levels in Ago2 immunoprecipitates relative to the IgG control group, confirming their co‐localization within the RNA‐induced silencing complex (RISC). *N* = 3 independent experiments per group. ***p < 0.001.

## Data Availability

The data that support the findings of this study are available from the corresponding author upon reasonable request.
