# Supplementary figures and images for: Circular RNA circ_0003423 Promotes Osteoarthritis Progression by Sponging miR‐330‐5p to Upregulate TWIST1‐Mediated Chondrocyte Inflammation and Extracellular Matrix Degradation
Source: J Cell Mol Med. 2026 Jul 6;30(13):e71278. doi: 10.1111/jcmm.71278 (PMC13337541; doi:10.1111/jcmm.71278)

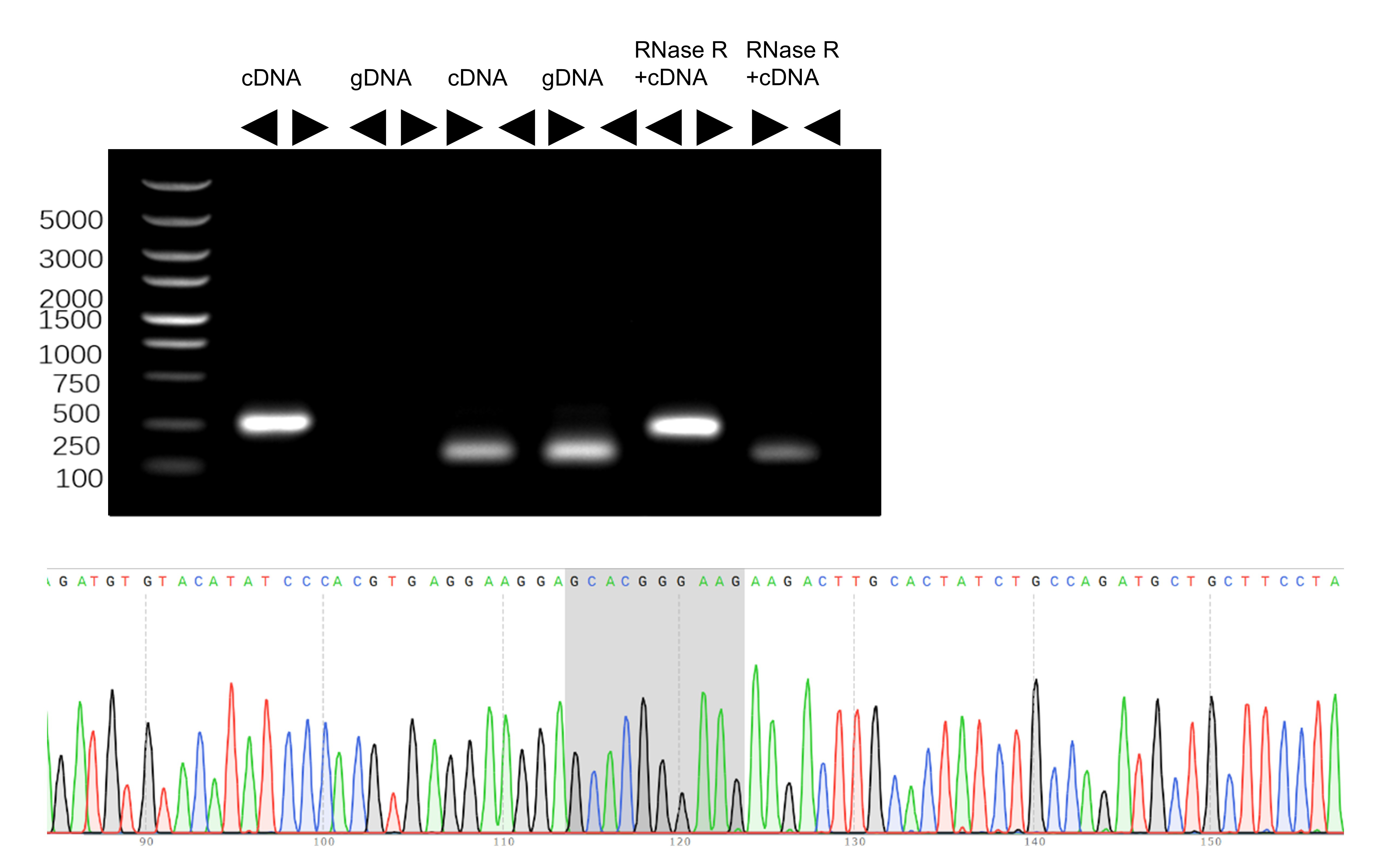

Supplement: Supplementary file 1 — Figure S1: Validation of the circular structure of circ_0003423. Complementary approaches were employed to confirm the circular nature of circ_0003423 in human chondrocytes: 1. PCR amplification using divergent and convergent primers with CDNA and genomic DNA (GDNA) as templates, with products resolved by agarose gel electrophoresis to confirm the existence of the back‐splice junction; 2. RNase R resistance assay in which total RNA was treated with or without RNase R, followed by PCR amplification using divergent and convergent primers to confirm resistance of circ_0003423 to linear RNA degradation; 3. Sanger sequencing chromatogram of the divergent primer‐amplified PCR product confirming the identity of the back‐splice junction of circ_0003423 upon alignment to the reference genome. [file JCMM-30-e71278-s002.JPG]

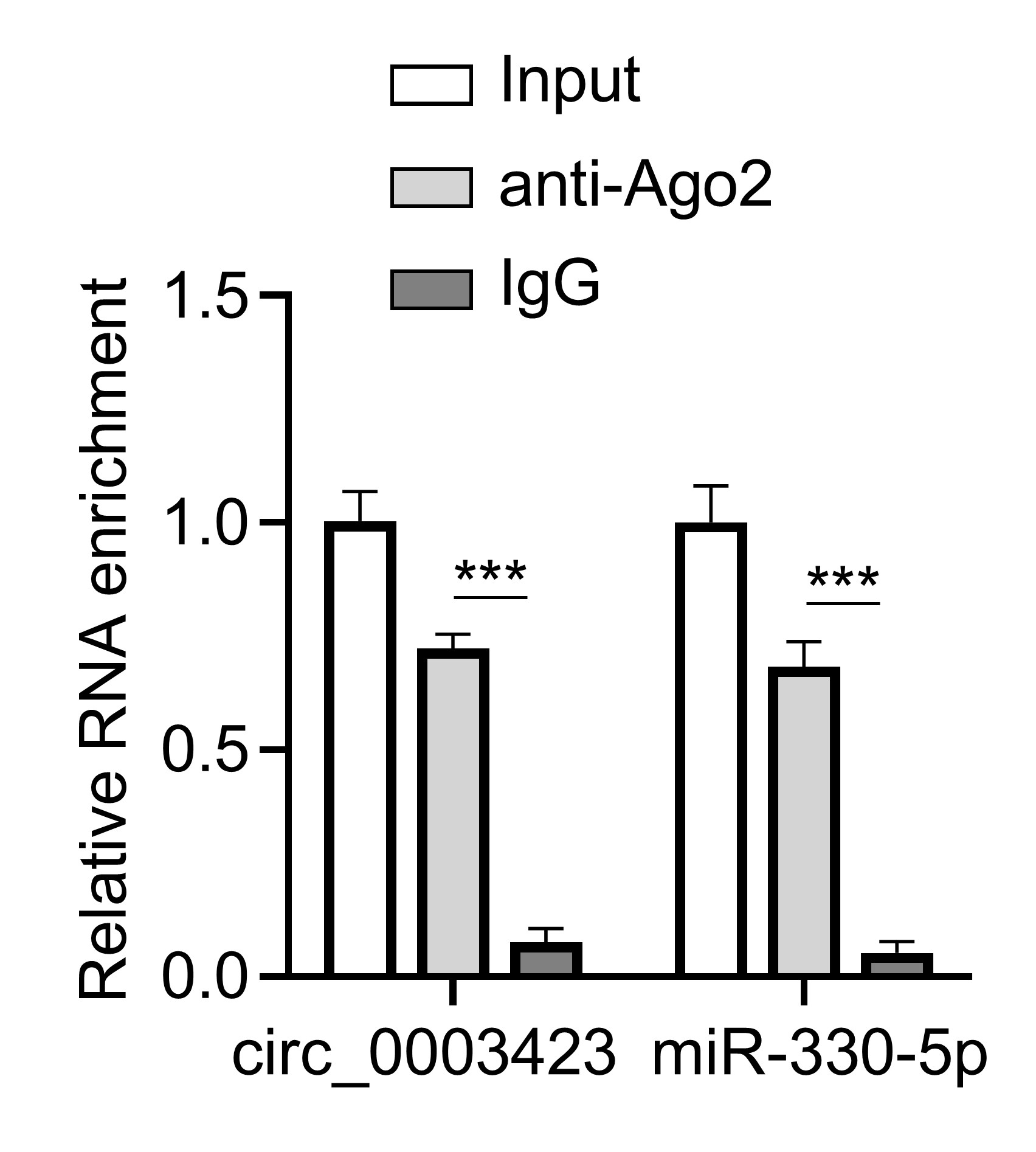

Supplement: Supplementary file 2 — Figure S2: RNA immunoprecipitation (RIP) assay confirms co‐enrichment of circ_0003423 and miR‐330‐5p within the RISC complex. Anti‐Ago2 RIP assay was performed in human chondrocytes using an anti‐Ago2 antibody or normal rabbit IgG as a negative control. QRT‐PCR quantification of circ_0003423 and miR‐330‐5p enrichment levels in Ago2 immunoprecipitates relative to the IgG control group, confirming their co‐localization within the RNA‐induced silencing complex (RISC). N = 3 independent experiments per group. ***p < 0.001. [file JCMM-30-e71278-s001.JPG]
